# Predictors of 30-day mortality and the risk of recurrent systemic thromboembolism in cancer patients suffering acute ischemic stroke

**DOI:** 10.1371/journal.pone.0172793

**Published:** 2017-03-10

**Authors:** Ki-Woong Nam, Chi Kyung Kim, Tae Jung Kim, Sang Joon An, Kyungmi Oh, Heejung Mo, Min Kyoung Kang, Moon-Ku Han, Andrew M. Demchuk, Sang-Bae Ko, Byung-Woo Yoon

**Affiliations:** 1 Department of Neurology, Seoul National University Hospital, Seoul, Korea; 2 Department of Neurology, Korea University Guro Hospital and Korea University College of Medicine, Seoul, Korea; 3 Calgary Stroke Program, Department of Clinical Neuroscience and Radiology, Hotchkiss Brain Institute, University of Calgary, Calgary, Canada; 4 Department of Neurology, Seoul National University Bundang Hospital, Seongnam, Korea; Duke University School of Medicine, UNITED STATES

## Abstract

**Background:**

Stroke in cancer patients is not rare but is a devastating event with high mortality. However, the predictors of mortality in stroke patients with cancer have not been well addressed. D-dimer could be a useful predictor because it can reflect both thromboembolic events and advanced stages of cancer.

**Aim:**

In this study, we evaluate the possibility of D-dimer as a predictor of 30-day mortality in stroke patients with active cancer.

**Methods:**

We included 210 ischemic stroke patients with active cancer. The 30-day mortality data were collected by reviewing medical records. We also collected follow-up D-dimer levels in 106 (50%) participants to evaluate the effects of treatment response on D-dimer levels.

**Results:**

Of the 210 participants, 30-day mortality occurred in 28 (13%) patients. Higher initial NIHSS scores, D-dimer levels, and CRP levels as well as frequent cryptogenic mechanism, systemic metastasis, multiple vascular territory lesion, hemorrhagic transformation, and larger infarct volume were related to 30-day mortality. In the multivariate analysis, D-dimer [adjusted OR (aOR) = 2.19; 95% CI, 1.46–3.28, *P* < 0.001] predicted 30-day mortality after adjusting for confounders. The initial NIHSS score (aOR = 1.07; 95% CI, 1.00–1.14, *P* = 0.043) and hemorrhagic transformation (aOR = 3.02; 95% CI, 1.10–8.29, *P* = 0.032) were also significant independent of D-dimer levels. In the analysis of D-dimer changes after treatment, the mortality group showed no significant decrease in D-dimer levels, despite treatment, while the survivor group showed the opposite response.

**Conclusions:**

D-dimer levels may predict 30-day mortality in acute ischemic stroke patients with active cancer.

## Introduction

Cancer and ischemic stroke are both leading causes of death worldwide. Stroke in cancer patients is not rare during its clinical course, present in up to 15% of patients, [1] but it is a devastating event with high mortality. [1, 2] Despite the high frequency of mortality, the predictors of this fatal outcome in stroke patients with cancer have not been fully evaluated because of the mixed pathophysiologic nature of cancer-related stroke. [3–5] However, D-dimer has appeared to be a useful indicator for the occurrence of cancer-related stroke in patients with active cancer because it may reflect the hypercoagulable state of cancer patients, including increased microembolic signals in brain arteries [6] and scattered small-sized embolic infarcts in brain magnetic resonance imaging (MRI). [7] In addition to being a predictor of the occurrence of thromboembolic events in cancer patients, D-dimer itself could be related to the prognosis, as it may be closely related to two major causes of death in cancer-related stroke: additional thromboembolic events and advanced stages of cancer. [8, 9]

The 30-day mortality rate is one of the important clinical outcomes for admitted patients with stroke. There have been several studies on the risk factors for 30-day mortality in ischemic stroke patients. [10–13] However, the importance of hypercoagulability on the 30-day mortality in stroke patients with cancer has not been addressed. In this study, we aimed to evaluate the possibility of applying D-dimer as a marker of hypercoagulability and progression of cancer to predict 30-day mortality in acute ischemic stroke patients with active cancer.

## Materials and methods

### Patients

We recruited a consecutive series of ischemic stroke patients admitted to two large stroke centers in Korea (Seoul National University Hospital and Seoul National University Bundang Hospital) within 7 days of symptom onset between March 2011 and June 2015 (n = 2820 and 4120). Of those, 261 patients had concurrent active cancer. Active cancer was defined as a diagnosis, recurrence, metastasis or progression within 6 months before enrollment. [6]

We excluded participants meeting the following criteria: younger than 18 years (n = 11), lack D-dimer data (n = 19), or show presence of a primary intracranial or hematologic malignancy, given their different mechanisms in stroke (n = 21). [14] Finally, a total of 210 patients were included in our study. This study was approved by the institutional review board at Seoul National University Hospital (IRB No. 1508-067-694). This study was designed as a retrospective study in which medical records were only reviewed. Thus, informed consent was not needed and even unattainable. Understanding of this problem, the IRB of Seoul National University Hospital approved this study, despite not having informed consent.

### Mortality data

The primary outcome in this study was 30-day mortality from any cause. The causes of death were evaluated by retrospectively reviewing medical records and classifying them by the primary mechanisms (e.g., myocardial infarction, pulmonary embolism, stroke recurrence, disseminated intravascular coagulation, brain herniation, infection) by neurologists who were not included in the current study. Stroke recurrence was defined as a fatal new stroke without correlation to the initial stroke lesion. Mortality caused by brain herniation was defined as a fatal herniation from an initial large stroke lesion.

### Clinical assessment

We collected baseline demographic and cardiovascular risk factors, including age, sex, hypertension, diabetes, hyperlipidemia, atrial fibrillation, current smoking status, [15] and venous thrombosis (Text in S1 Appendix). Data related to cancer, including cancer type, systemic metastasis, brain metastasis, and proportion of adenocarcinoma were also evaluated. All evaluations regarding cancer were performed by pathologic confirmation by oncologists.

The initial clinical data were assessed, including initial stroke severity, stroke mechanism, body temperature, blood pressure, and use of anti-thrombotic agents and thrombolytic therapy. We evaluated the stroke mechanisms using the Trial of Org 10172 in Acute Stroke Treatment (TOAST) classification. Then, we divided the classifications into conventional (large artery atherosclerosis, cardioembolism, small vessel occlusion) and cryptogenic mechanisms. Stroke severity was assessed by formally trained neurologists using the NIH stroke scale (NIHSS) score. All participants underwent routine blood tests, and coagulation studies, including measurement of D-dimer, and C-reactive protein (CRP) levels, electrocardiography (ECG) and echocardiography. D-dimer levels were evaluated within 24 hours of admission.

### Radiological evaluation

All participants underwent MRI and magnetic resonance arteriography (MRA) within 24 hours of admission using a 3.0 T MR scanner (Achieva 3.0 T; Philips, Eindohoven, the Netherlands). MRI lesions were divided into two groups: single vascular territory lesions and multiple vascular territory lesions, as multiple territory lesions are thought to be related to hypercoagulability in ischemic stroke patients with active cancer. [7, 16, 17] We calculated the initial infarct volume on diffusion-weighted images (DWI) using Medical Imaging Processing, Analysis, and Visualization (MIPAV, version 7.3.0, National Institutes of Health, Bethesda, MD). We also evaluated hemorrhagic transformation, ranging from hemorrhagic infarction to petechial hemorrhage. [18]

### Statistical analysis

We presented normally distributed data as the mean ± SD, while non-normally distributed data were presented as the median ± IQR. All skewed variables were transformed to log-scale for statistical analyses. In univariate analysis, Student’s t-test or Mann-Whitney *U*-test was used for continuous variables. The chi-squared test or Fisher’s exact test was used for categorical variables. The variables with multiple categorical values were analyzed using a linear-by-linear association method. In the multivariate analyses, we used binary logistic regression analysis to evaluate the relationship between 30-day mortality and D-dimer levels, including all variables found to have *P* < 0.05 in the univariate analysis and initial thrombolytic therapy as confounders.

To analyze the responses after treatment, we selected participants who had follow-up D-dimer levels (n = 106). The Wilcoxon-rank test was used to evaluate the differences between the initial and post-treatment D-dimer levels in the both mortality and the survivor group. Then, we divided the participants into responders and non-responders. Non-responders were defined as participants who had equal to increased follow-up D-dimer levels, despite treatment. The others were classified as responders. All values of *P* < 0.05 were considered statistically significant. All statistical analyses were conducted by the Statistical Package for the Social Sciences (SPSS version 22.0; IBM, Chicago, IL, USA).

## Results

**Fig 1:**
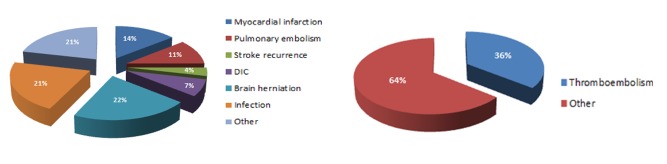
Causes of death in the 30-day mortality group in ischemic stroke with active cancer.

We included a total of 210 ischemic stroke patients (mean age = 68 years, initial NIHSS score = 4 [2–11], time delay from onset to visit = 7 [2–24] hours) with active cancer. 30-day mortality was detected in 28 (13%) patients. The main causes of death were as follow: 4 myocardial infarctions (14%), 3 pulmonary embolisms (11%), 1 stroke recurrence (4%), 2 disseminated intravascular coagulation (7%), 6 brain herniations (21%), 6 infections (21%), and 6 other causes (21%; e.g., tumor bleeding, metabolic acidosis, malignant effusion, and obstructive jaundice) (Fig 1).

We evaluated the differences in demographic, clinical, laboratory and radiological factors between those with and without 30-day mortality (Table 1, S1 Table and S2 Table). Higher initial NIHSS scores, D-dimer levels, and CRP levels as well as frequent cryptogenic mechanism, systemic metastasis, multiple vascular territory lesions, hemorrhagic transformation, and larger infarct volume were related to 30-day mortality.

**Table 1:** Baseline characteristics of patients with and without 30-day mortality.

	Survivor (n = 182)	Mortality (n = 28)	*Ρ*
Hospitalization duration, d [IQR]	11 [8–20]	13 [7–21]	0.772
Age, y [IQR]	70 [63–75]	68 [55–81]	0.707
Sex, Male%	106 (58)	16 (57)	0.913
Stroke mechanism, %			0.030
Conventional	71 (39)	5 (18)	
Cryptogenic	111 (61)	23 (82)	
Venous thrombosis, %	25 (14)	4 (14)	1.000
Cancer type, %			0.328
Lung	53 (29)	12 (43)	
Stomach	23 (13)	2 (7)	
Colorectal	14 (8)	0 (0)	
Hepatobiliary	52 (29)	10 (36)	
Breast	5 (3)	1 (4)	
Genitourinary	18 (10)	1 (4)	
Prostate	10 (5)	1 (4)	
Other	7 (4)	1 (4)	
Systemic metastasis, %	137 (76)	26 (93)	0.049
Brain metastasis, %	17 (9)	5 (18)	0.171
Adenocarcinoma, %	116 (69)	21 (81)	0.208
Initial NIHSS [IQR]	4 [2–9]	12 [4–18]	0.001
Initial BT, °C [IQR]	36.7 [36.4–36.9]	36.8 [36.5–37.3]	0.089
Infarct volume, mL [IQR]	4.29 [1.24–14.72]	34.28 [6.06–83.52]	< 0.001
D-dimer, μg/mL [IQR]	2.17 [0.95–8.93]	14.53 [4.73–20.00]	< 0.001
WBC, x10^3^/mL [IQR]	7.50 [5.80–10.80]	9.27 [7.06–11.25]	0.098
CRP, mg/dL [IQR]	1.37 [0.15–6.11]	6.49 [1.83–15.70]	< 0.001
MRI pattern, %			0.004
Single territory	99 (54)	7 (25)	
Multiple territory	83 (46)	21 (75)	
Hemorrhagic transformation, %	31 (17)	10 (36)	0.020
Initial anti-thrombotic, %			0.267
Anti-coagulation	103 (57)	17 (61)	
Anti-platelet	68 (37)	8 (29)	
Combined	8 (4)	0 (0)	
No medication	3 (2)	3 (11)	
Thrombolytic therapy, %			0.760
Intravenous	5 (3)	2 (7)	
Intraarterial	9 (5)	3 (11)	
Both	7 (4)	0 (0)	
None	161 (88)	23 (82)	

NIHSS = National Institutes of Health Stroke Scale, BT = Body temperature, WBC = White blood cell, CRP = C-reactive protein

In the multivariate analysis, D-dimer levels remained an independent predictor of 30-day mortality [Adjusted OR (aOR) = 2.19; 95% CI, 1.46–3.28, *P* < 0.001, Table 2]. Initial NIHSS score (aOR = 1.07; 95% CI, 1.00–1.14, *P* = 0.043) and hemorrhagic transformation (aOR = 3.02; 95% CI, 1.10–8.29, *P* = 0.032) were also significant, independent of D-dimer levels. MRI pattern and cryptogenic mechanism were not included in the multivariate analysis, considering their severe correlation with D-dimer levels (Pearson correlation analysis *P* < 0.001 for both).

**Table 2:** Multivariate analysis of possible predictors of 30-day mortality.

	Crude OR	*P*	Adjusted OR	*P*
D-dimer	2.12 [1.48–3.04]	< 0.001	2.19 [1.46–3.28]	< 0.001
CRP	1.12 [1.05–1.19]	< 0.001	…	…
Initial NIHSS	1.11 [1.05–1.17]	< 0.001	1.07 [1.00–1.14]	0.043
Infarct volume	1.61 [1.24–2.09]	< 0.001	…	…
Systemic metastasis	4.18 [0.95–18.30]	0.058	…	…
Hemorrhagic transformation	2.71 [1.14–6.42]	0.024	3.02 [1.10–8.29]	0.032
Thrombolytic therapy	1.67 [0.57–4.85]	0.349	…	…

CRP = C-reactive protein, NIHSS = National Institutes of Health Stroke Scale

**Fig 2:**
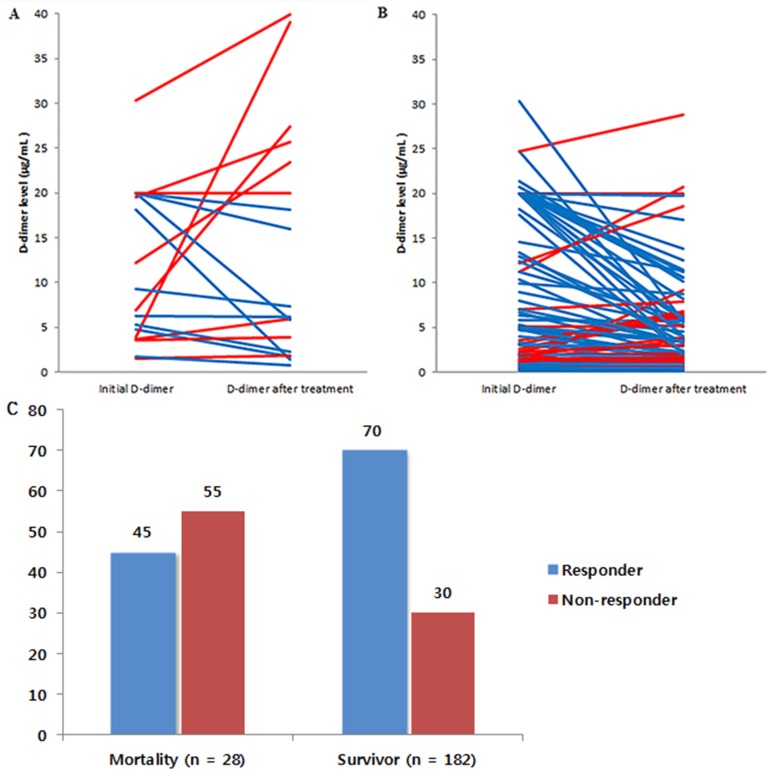
Changes in D-dimer levels from initial levels and those after treatment. Changes in D-dimer levels after treatment in the mortality group (A) and the survivor group (B). Red lines represent non-responders (no change or incremental responses), and blue lines represent responders (decremental responses). Responders were more frequent in the mortality group than the survivor group (C).

We also analyzed the responses of D-dimer levels after treatment in those with and without 30-day mortality. In this analysis, 106 (50%) participants had follow-up D-dimer levels (71% in the mortality group and 47% in the survivor group). The follow-up duration of D-dimer levels was 4 [2–9] days. Initial D-dimer levels (10.77 [4.34–20.00] versus 4.82 [1.37–18.20], *P* = 0.039) and follow-up D-dimer levels (11.66 [3.12–21.70] versus 3.34 [1.12–6.55], *P* = 0.002) were higher in the mortality group. The responses of the D-dimer levels after treatment are presented in Fig 2. In the mortality group, only 45% of the patients showed responses despite treatment (Wilcoxon-rank test, *P* = 0.831). In contrast, D-dimer levels declined after treatment in 70% of the survivor group (Wilcoxon-rank test, *P* < 0.001).

In the univariate analysis that evaluated differences between D-dimer responders and non-responders to treatment, the non-responders showed frequent 30-day mortality and brain metastasis (Table 3). There were marginal trends of more frequent systemic metastasis and less use of anti-coagulation. The major causes of death in patients defined as non-responders were systemic thromboembolisms in 55% (including 9% DIC, 27% pulmonary embolism, and 18% myocardial infarction), while no one of the responders died due to systemic thromboembolism. (S1 Fig)

**Table 3:** Characteristics of responders and non-responders.

	Non-responder (n = 37)	Responder (n = 69)	*Ρ*
30-day mortality, %	11 (30)	9 (13)	0.036
D-dimer follow-up, day [IQR]	5 [3–9]	5 [3–7]	0.612
Cancer type, %			0.780
Lung	15 (41)	18 (26)	
Stomach	1 (3)	11 (16)	
Colorectal	2 (5)	3 (4)	
Hepatobiliary	12 (32)	26 (38)	
Breast	2 (5)	1 (1)	
Genitourinary	2 (5)	7 (10)	
Prostate	2 (5)	2 (3)	
Other	1 (3)	1 (1)	
Systemic metastasis, %	30 (81)	63 (93)	0.075
Brain metastasis, %	6 (16)	3 (4)	0.037
Initial NIHSS [IQR]	5 [3–13]	4 [2–9]	0.335
Infarct volume, mL [IQR]	8.63 [1.53–40.13]	9.05 [1.96–20.99]	0.472
D-dimer, μg/mL [IQR]	3.53 [1.37–12.20]	6.67 [1.95–20.00]	0.169
Use of anti-coagulation	23 (62)	54 (78)	0.076
Initial anti-thrombotic, %			0.105
Anti-coagulation	23 (62)	54 (78)	
Anti-platelet	12 (32)	14 (20)	
Combined	1 (3)	1 (1)	
No medication	1 (3)	0 (0)	
Thrombolytic therapy, %			0.590
Intravenous	1 (3)	1 (1)	
Intraarterial	2 (5)	4 (6)	
Both	2 (5)	2 (3)	
None	32 (86)	62 (90)	

NIHSS = National Institutes of Health Stroke Scale

## Discussion

In this study, we found that 30-day mortality occurred in 13% of our participants. We also found that D-dimer was an independent predictor of 30-day mortality in ischemic stroke patients with active cancer.

The elevated D-dimer levels in the mortality group could be the result of several causes. First, systemic thromboembolism due to hypercoagulability in cancer patients may be important. We found that systemic thromboembolism was responsible for 36% of the cases in the mortality group, which showed almost twice as many multiple vascular territory lesion cases. Moreover, we also found a higher proportion of cryptogenic stroke in the mortality group (82%), which was believed to be more related to hypercoagulability. [6, 7] Thus, systemic thromboembolism might be a mechanism of 30-day mortality, and elevated D-dimer levels may reflect an increased risk of systemic thromboembolism by hypercoagulability. Second, D-dimer could be elevated as a reflection of large volume infarctions. In this study, D-dimer levels were also high in those with severe and large stroke, consistent with previous studies. [19] Because 22% of the mortality group died as a result of brain herniation after large volume infarction, elevated D-dimer might be an indicator of large volume infarctions that have a poor prognosis. Third, high D-dimer levels may suggest more advanced cancer itself. Systemic metastasis and cancer progression have been observed to be correlated with D-dimer levels. [8, 9] Baseline general conditions caused by more advanced cancer may lead to worse outcomes.

An additional finding in this study was related to the changes in D-dimer levels after treatment. In the analysis of 106 participants with follow-up D-dimer levels, the mortality group showed a higher frequency of non-responders. These non-responders seemed to be correlated with brain metastasis, showing marginal trends of association with systemic metastasis and no use of anti-coagulation. Thus, advanced cancer status and use of anti-coagulation might be related to changes in D-dimer levels in these patients. In the analysis of causes of death in both responders and non-responders, non-responders more frequently died from systemic thromboembolism. Thus, we carefully suggest that providing more intensive use of anti-coagulation and monitoring follow-up D-dimer levels in stroke patients with advanced cancer may be considered.

There are some caveats to these findings. First, this is a retrospective study based on data from two centers. Thus, selection bias and the possibility of overfitting may have existed. Second, we identified follow-up D-dimer levels in only 50% of the participants with variable follow-up durations. Because we checked follow-up D-dimer levels more frequently in cases with worse clinical courses, the results should be interpreted cautiously. However, because persistently increased D-dimer levels could be a marker of a more thrombogenic condition or larger tumor burden, it is conceptually reasonable that we postulated that non-responders defined by D-dimer levels after treatment had poor outcomes. To clarify this issue, further prospective studies with larger sample sizes are needed.

In conclusion, D-dimer levels may predict 30-day mortality in acute ischemic stroke patients with active cancer. Close monitoring and follow-up of D-dimer levels is warranted in this group, and further large studies are needed to confirm these results.

## Supporting information

S1 Appendix Definition of clinical risk factors. (DOCX)

S1 Fig Major causes of death in responder and non-responder. Major causes of death in non-responder (A) were systemic thromboembolism in 55% (18% myocardial infarction, 28% pulmonary embolism, and 9% DIC), while no one of the responders (B) died due to systemic thromboembolism.
(TIF)

S1 Table Distributions of initial treatment. (DOCX)

S2 Table Cardiovascular risk factors between with and without 30-day mortality. (DOCX)
