# Supplementary material for: Predictors of 30-day mortality and the risk of recurrent systemic thromboembolism in cancer patients suffering acute ischemic stroke
Source: PLoS One. 2017 Mar 10;12(3):e0172793. doi: 10.1371/journal.pone.0172793 (PMC5345775; doi:10.1371/journal.pone.0172793)
Supplement: S1 Appendix — (DOCX) [file pone.0172793.s001.docx]

S1 Method Definition of clinical risk factors

1. Hypertension:
   1. Use of anti-hypertensive agent
   2. Systolic blood pressure > 140 mmHg at discharge
   3. Diastolic blood pressure > 90 mmHg at discharge
2. Diabetes:
   1. Use of glucose-lowering agent
   2. Hemoglobin A1c ≥ 6.5%)
3. Hyperlipidemia:
   1. Use of lipid-lowering agent
   2. Fasting low-density lipoprotein cholesterol >160 mg/dl
   3. Fasting total cholesterol > 240 mg/dl
4. Venous thrombosis:
   1. Deep vein thrombosis, or pulmonary embolism which was clinically or incidentally found
