# Supplementary figures and images for: Predictors of 30-day mortality and the risk of recurrent systemic thromboembolism in cancer patients suffering acute ischemic stroke
Source: PLoS One. 2017 Mar 10;12(3):e0172793. doi: 10.1371/journal.pone.0172793 (PMC5345775; doi:10.1371/journal.pone.0172793)

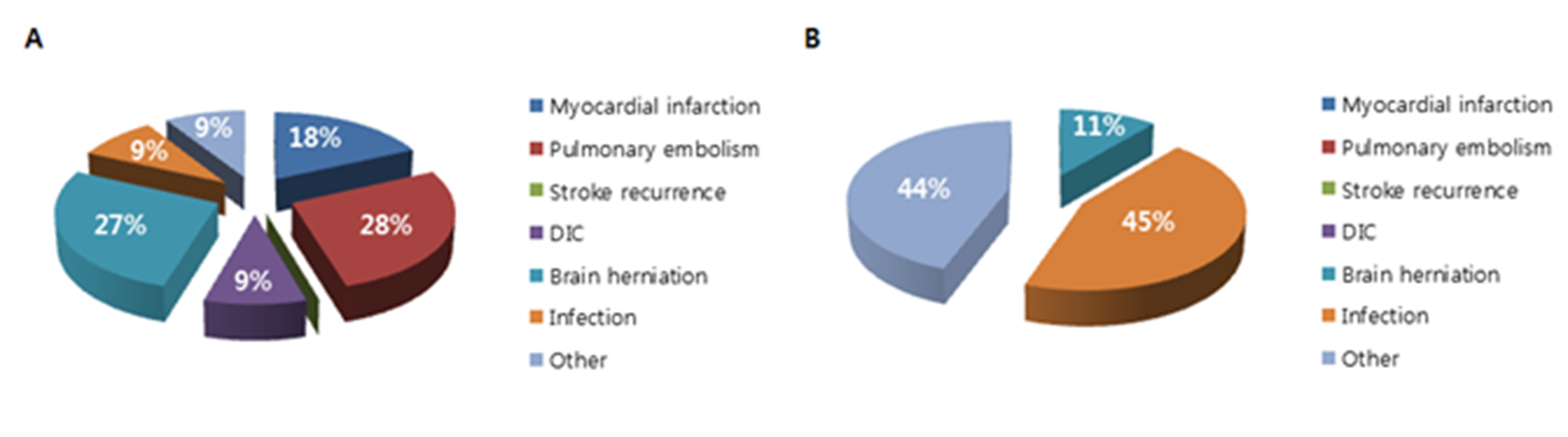

Supplement: S1 Fig — Major causes of death in non-responder (A) were systemic thromboembolism in 55% (18% myocardial infarction, 28% pulmonary embolism, and 9% DIC), while no one of the responders (B) died due to systemic thromboembolism. (TIF) [file pone.0172793.s002.tif]
