# Supplementary material for: Predictors of 30-day mortality and the risk of recurrent systemic thromboembolism in cancer patients suffering acute ischemic stroke
Source: PLoS One. 2017 Mar 10;12(3):e0172793. doi: 10.1371/journal.pone.0172793 (PMC5345775; doi:10.1371/journal.pone.0172793)
Supplement: S1 Table — (DOCX) [file pone.0172793.s003.docx]

S1 Table S1 Distributions of initial treatment

|  | Survivor (n = 182) | Mortality (n = 28) | *Ρ* |
| --- | --- | --- | --- |
| Initial anti-thrombotic, % |  |  | 0.267 |
| Anti-coagulation | 103 (57) | 17 (61) |  |
| Anti-platelet | 68 (37) | 8 (29) |  |
| Combined | 8 (4) | 0 (0) |  |
| No medication | 3 (2) | 3 (11) |  |

Distributions of anti-coagulant between two groups

|  | Survivor (n = 103) | Mortality (n = 17) | *Ρ* |
| --- | --- | --- | --- |
| Anti-coagulant, % |  |  | 0.347 |
| Low-molecular weight heparin | 83 (81) | 12 (71) |  |
| Oral anti-coagulant | 20 (19) | 5 (29) |  |
