# Supplementary material for: Predictors of 30-day mortality and the risk of recurrent systemic thromboembolism in cancer patients suffering acute ischemic stroke
Source: PLoS One. 2017 Mar 10;12(3):e0172793. doi: 10.1371/journal.pone.0172793 (PMC5345775; doi:10.1371/journal.pone.0172793)
Supplement: S2 Table — (DOCX) [file pone.0172793.s004.docx]

S2 Table Cardiovascular risk factors between with and without 30-day mortality

|  | Survivor (n = 182) | Mortality (n = 28) | *Ρ* |
| --- | --- | --- | --- |
| Hypertension, % | 92 (51) | 12 (43) | 0.449 |
| Diabetes, % | 50 (27) | 4 (14) | 0.167 |
| Hyperlipidemia, % | 27 (15) | 4 (14) | 1.000 |
| Atrial fibrillation, % | 20 (11) | 4 (14) | 0.536 |
| Current smoking, % | 37 (20) | 3 (11) | 0.305 |
| Initial SBP, mmHg [SD] | 132 ± 22 | 134 ± 23 | 0.811 |
| Initial DBP, mmHg [IQR] | 74 [67-82] | 78 [70-85] | 0.202 |
